# The Hospital Crisis: A Dangerous Misapprehension

**Published:** 1919-12-06

**Authors:** 


### The Hospital

The Workers' Newspaper of Administrative Medicine and Institutional Life, Administration, National Insurance and Health.

No. 1748, Vol. LXVII. SATURDAY, DECEMBER 6, 1919. PRICE TWOPENCE

### THE HOSPITAL CRISIS: A DANGEROUS MISAPPREHENSION.

In last week's issue we showed how all the money required by the voluntary hospitals can be obtained with relative ease and assured certainty. So imperfectly is the art of raising funds for the voluntary hospitals understood by many hospitals, and so readily has money come to most of them from the public in sufficient quantities to keep the doors open in the past, that a large number of institutions possess in a business sense no organised and permanent machinery worthy of the name devoted to finance and the raising of money. The voluntary system after the war must undergo considerable modification, so far as the ground covered by each institution and the accommodation it provides are concerned. The ground to be covered includes accommodation under the new regulations and an up-to-date system which will provide for all classes of the community who when seriously ill or requiring important operative measures seek accommodation in the voluntary hospital as the surest and best method to adopt.

The voluntary hospital in these days has extended its area of work, and has blossomed into a great all-embracing cure-house where treatment is provided for every type of ailment to which the human frame is liable. As recently as twenty-five years ago the number of separate departments included in the organisation of a great voluntary hospital did not exceed a round dozen, if so many. To-day the larger hospitals—and, indeed, some of the more ambitious of the smaller ones—have their special departments, including some or all of the following: Medical, surgical, aural, gynæcological, ophthalmic, skin, dental, radiological, physical treatment, light, Finsen light, inoculation, heart, pathological laboratory, clinical laboratory, bacteriological laboratory, venereal, "follow-through," and children's diseases, *i.e.*, nineteen departments in 1918 compared with three departments in 1893. These departments have entailed a large increase in the staff of the medical, dispensing, and other departments. In one great hospital the salaries in these departments have collectively increased in the twenty-five years from roughly £8,000 to £26,500. The in-patients have increased from 9,945 to 16,053, and the average residence of patients, which was 24.4 days in 1893, has been reduced to 18.9 in 1918. The ordinary expenditure at the great hospital in question in 1913, an average normal year, was £118,300. In 1918, an abnormal year in every way because of the war, it increased to £200,200, an increase of 69 per cent. on the normal year.

The foregoing facts, which are not understood by the public or grasped by the majority of those who support the hospitals, represent in practice great changes, entailing, as the figures we quote show, an increase in the actual expenditure by the best administered hospitals throughout the country, which have honourably responded to the needs of the population, the advancement of science, and the introduction of up-to-date methods of treatment whereby life has been immeasurably prolonged, the sufferings of the patients materially decreased, and the general health of the community raised to a standard never before attained in these islands. How, then, has it been possible to keep the hospitals efficient and to equip them with adequate funds, impedimenta for service, new and expensively equipped departments, and all the other requisites now available to diminish suffering, lessen the days' residence in the hospitals, and restore patients speedily to health, and under conditions which place them in the first rank of successful breadwinners and maintainers of households and families? The first reason is that the magnificence of the work and opportunity has attracted to our voluntary hospital system a type of voluntary workers who have become members or chairmen of committees and have devoted wholeheartedly much time to the service of the community through the hospitals to which they have attached themselves. Representing, as many of them do, the leading firms and acutest minds in the business and industrial worlds, they have through their service first awakened and then secured the interest of some of the most wealthy members of the community which the hospitals in question serve; and, being large employers of labour, and the best of masters many of them, the working classes have in the last twenty years been brought to know and realise what has been going on within the hospitals for the benefit of themselves and their families. Thus the sums at present given to the voluntary hospitals by the working classes are greater than they have ever been in their history.

Such has been the internal condition of the most successfully administered voluntary hospitals up to the Armistice. In the last twelve months especially there has been a thorough upset of the minds, habits, and outlook of many classes of the people in these islands. This has been followed by unrest, dissatisfaction, and the extended spread of the spirit of discontent which has been fomented by unwholesome and unsound propaganda on the part of agitators and misleaders of the people, including a number of aliens of the Bolshevist type, who are fed by funds supplied from abroad. These agitators aim at arousing among the masses of the people dangerous feelings of discontent with their surroundings and outlook, which the unthinking are taught to believe can be changed by the use of State funds in substitution for a wholesome condition of honest work for honest men and women, producing economic changes adequate to keep the home fires burning, and to make the home a centre of happy, healthy, wholesome life. Not the least harmful of these agitators represent a type among the working classes which has started these herrings: First, that the voluntary system of hospitals is doomed financially, and, secondly, that the only way, and the best, for the people who frequent the hospitals is to put the latter on the rates. We have recently published striking evidence of the miseries and loss which may result to the working classes if the whole of our voluntary hospitals were to be swept away and rate-supported hospitals substituted. At present the working men and women have not realised the dangers with which they are threatened by the propaganda of putting the hospitals on the rates. The treasurers of our voluntary hospitals, however, have already begun to have startling evidence of the dangers, which have resulted in loss to the revenues of the voluntary hospitals. Space will only permit us to take this point to-day and explain the reason.

The ablest of the citizens who are wholeheartedly desirous to make the lot of every person in the United Kingdom, however humble their circumstances, secure and happy, who, as we have explained, have been and are at the moment largely the financial backbone on which the voluntary hospital system rests, realise what a change in efficiency, what a dreadful setback to progress and the uplifting and maintenance of the health of the people, would result if the voluntary hospital system were abolished and all these institutions had to rely on State support for their upkeep and continuance. During the last few weeks we have had intimations of the actual change in the attitude of hospital supporters which is contemplated by some of the very best of them, because they are old enough and have taken a sufficiently active interest in local affairs in this country to judge for themselves the degeneracy in system which must ensue if the noble hospitals of to-day are going to be State-owned and State-administered. This is a matter which calls for the immediate attention of the Prime Minister and his colleagues in the Government. Dr. Addison, the Minister of Health, is credited with being of opinion that State-owned and State-administered hospitals throughout the country are ideals which he would like to further and uphold. We believe, from inquiries we have made, and taking some utterances of his at their face value, that Dr. Addison recognises, as all statesmen must recognise, that the State has so much on its hands that any question of the nationalisation of hospitals, apart altogether from the evils and merits, if any, of such a system, must be postponed into the very dim future. We therefore appeal to the Government, and to Dr. Addison especially, to take an early opportunity to make this, the actual position of matters, clear to the nation and people of these islands, that the agitation for placing our voluntary hospitals on the rates may be promptly checked.

**The danger of the agitation in the financial sense to the hospitals is demonstrably provable. Here are some facts within our knowledge. A liberal supporter of an important county hospital stopped giving the contribution which he had annually sent to its treasurer. On investigating the matter, it was found that the reason he had suspended his gifts to the hospital was that the Government were going to waste money, at the instance of working-class agitators, by letting the hospitals go on the rates. He held, and a number of other supporters joined in the plea, that the result in the near future must be that our splendid hospitals would be degenerated to the workhouse level. A number of hospitals which rely upon the revenue derived from legacies have become aware that the makers of wills are suspending their legacies to hospitals until they have the assurance that the Government are not going to place them on the rates, and so cause them to become State-owned and State-administered.**

### A Word for the Ministry of Health.

Arising from this disturbance threatened to the steady and heretofore adequate support of our voluntary hospitals there is added another danger which for hygienic and just reasons should be removed forthwith. The pressure on the hospitals for beds and treatment is greater to-day than it was before the war. A number of the hospitals have erected temporary pavilions in their grounds, where they have provided accommodation for wounded, septic, and poisoned cases of soldiers sent home from the Front. The hygienic arrangements in several of these cases were temporary to dangerous inefficiency, and, as hospital sites are limited in area, the continuance of these buildings in juxtaposition to the hospital buildings proper has become in some cases a danger to the health of the inmates of hospital beds. The health officers throughout the country should inspect every one of these temporary erections and give instructions for their removal and the scrapping of the material of which they are made. As it is at present some hospitals, pressed for funds and impelled to find accommodation somewhere for the many applicants who urgently demand admission and treatment, either contemplate or have actually allowed some of these insanitary temporary pavilions to be occupied by healthy civilian patients. All these matters are of the first importance to the health of the whole nation, and we venture to hope that the Ministry of Health will lose not a moment in taking such action as will demonstrate to the whole nation how important it is to public health that this new Department should be brought into being and efficiently administered throughout the country.

